# Strictosidine Synthase-like Gene *NMS1* Is Required for Male Fertility in Rice by Regulating Tapetal Degradation

**DOI:** 10.3390/genes17080970

**Published:** 2026-08-19

**Authors:** Zhiyuan He, Anping Du, Nenggang Chen, Yulin Tang, Ping Wang, Longxiang Lu, Hui Li, Yulu Bai, Shicong Yu, Jing Liang, Xiaoqing Yan, Lei Zhou, Xiaolan Liu, Zhigang Pu, Binhua Hu

**Affiliations:** 1 Institute of Biotechnology and Nuclear Technology, Sichuan Academy of Agricultural Sciences, Chengdu 610066, China; hezy1205@163.com (Z.H.); 18892891705@163.com (Y.T.); wangping2014@163.com (P.W.); baiyulu2007@126.com (Y.B.); shicongyu@aliyun.com (S.Y.); 18202890753@163.com (J.L.); antiant@163.com (L.Z.); lxl1633421@163.com (X.L.); zhigangpu@126.com (Z.P.); 2 Chengdu Institute of Biology, Chinese Academy of Sciences, Chengdu 610041, China; llxdewyyx@163.com (L.L.); lihui@cib.ac.cn (H.L.); 3 Institute of Crop Germplasm Resources, Guizhou Academy of Agricultural Sciences, Guiyang 550006, China; chennenggang123@163.com (N.C.); yxq201512252026@163.com (X.Y.)

**Keywords:** rice, male sterility, tapetal degradation, strictosidine synthase-like gene, transcriptome sequencing

## Abstract

**Background:** Male sterility is a critical trait for large-scale hybrid rice seed production, yet the genetic and molecular regulatory networks governing tapetal degradation during anther development remain incompletely understood. This study aimed to clone the causal gene underlying a novel rice non-pollen male sterility mutant and elucidate its role in tapetal development and microsporogenesis. **Methods:** The *nms1* (*non-pollen male sterility 1*) sterile mutant was screened from the ethyl methanesulfonate (EMS)-mutagenized progeny of the elite *indica* restorer line Shuhui 498 (R498). Map-based cloning and whole-genome resequencing-assisted bulked segregant analysis were used to identify the causal variant. Gene function was verified via cytological observation, genetic complementation testing, RNA sequencing, and quantitative real-time PCR (qRT-PCR) to profile sterility-associated transcriptional changes. **Results:** Gene mapping identified a T635A single-nucleotide substitution within *OsR498G0305626400.01* on chromosome 3, which encodes a strictosidine synthase-like protein. This nucleotide alteration causes a Val212Glu amino acid change and is associated with delayed tapetal degradation and pollen abortion. Transgenic complementation experiments verified that functional *NMS1* restores fertility in *nms1* mutant plants. Spatiotemporal expression analysis showed predominant *NMS1* expression in late-developing spikelets. Furthermore, combined RNA sequencing and qRT-PCR analyses demonstrated that loss of *NMS1* function leads to significant transcriptional dysregulation of key regulators of programmed cell death (PCD) in the tapetum (*PTC2*, *TIP2*) and pollen wall biosynthesis genes (*TIP3*, *OsMS2*). **Conclusions:** This study demonstrates that *NMS1* plays a crucial role in coordinating tapetal degradation and microspore development in rice. The discovered functional SNP of *NMS1* provides a novel theoretical foundation and a valuable sterile genetic resource for hybrid rice breeding.

## 1. Introduction

Rice (*Oryza sativa*) is one of the most vital staple crops worldwide, providing food for over half of the global population. Its productivity is therefore crucial to global food supply and security [1]. Heterosis, the superior performance of hybrid offspring over their parents, has been widely exploited in plant breeding to improve crop yield and stress tolerance [2]. Hybrid rice breeding has delivered substantial increases in grain yield and is currently widely cultivated across major rice-producing regions in Asia. Notably, the utilization of male-sterile lines constitutes the cornerstone of hybrid breeding systems, as it not only enables the efficient large-scale production of hybrid seeds but also accelerates the development of rice varieties with synergistic improvements in yield and quality.

The rice stamen comprises six anthers, and their development is a precisely regulated process involving several stages: anther primordium differentiation, specialization of anther wall cells, formation of pollen mother cells, meiosis, microspore development, programmed degradation of tapetal cells, and ultimately anther dehiscence to release pollen [3]. Pollen development comprises a series of key events, including the division and differentiation of pollen mother cells, meiosis, programmed cell death (PCD) of the tapetum, microspore development, and pollen wall formation. This developmental sequence depends on the coordinated expression of numerous genes, and disruption of any critical regulator can result in male sterility [4]. Abnormal tapetum development is a common cause of male sterility in rice. The tapetum plays essential roles in supplying nutrients for microspore development and precursors for pollen wall formation [5]. The precisely timed initiation of tapetal PCD is crucial for male fertility, as both premature and delayed onset can lead to male sterility in rice [6]. Tapetum development relies on a network of genes, and defects in any component can disrupt cellular development or PCD, ultimately causing microspore abortion. Some key genes involved in regulating rice tapetum development that have been cloned include *UDT1* [7], *OsTDF1* [8], *TDR* [9], *OsMS188* [10], *PTC1* [11], *OsMADS3* [12], *OsMADS8* [13], *EAT1* [14], *OsGAMYB* [15] *MSP1* [16], *OsUGE1* [17], *TIP2* [18], *TIP3* [19], *OsAGO2* [20], *EDT1* [21], *DTC1* [22], *SAW1* [23], and *OsEDM2L* [24]. For example, *UDT1* encodes a nuclear protein essential for normal tapetum and microspore development during meiosis [7]. Mutations in *ostdf1* cause vacuolation and hypertrophy of tapetal cells, resulting in complete male sterility [8]. *TDR* encodes a bHLH transcription factor that regulates tapetal PCD, and the *tdr* mutants display delayed tapetal degradation and abnormal microspore mother cells [9]. *OsMS188*, an MYB transcription factor, also modulates tapetal PCD [10]. *PTC1* encodes a PHD-finger protein critical for PCD and pollen development [11]. *OsMADS3* and *OsMADS8*, both MADS-box transcription factors, are essential for late anther development, with mutants showing aberrant tapetum, microspore degeneration, and sterility [12,13]. *EAT1* is a bHLH factor that promotes tapetal PCD [14], whereas *Osgamyb*, an R2R3 MYB mutant, exhibits delayed tapetum degradation and sterility [15]. Mutants, such as *osuge1*, *tip2*, *tip3*, and *osedm2l*, all exhibit delayed tapetal PCD and complete sterility [17,18,19,24]. *OsAGO2* is expressed in the tapetum during microspore development, and its knockout leads to excess ROS and premature PCD [20]. *EDT1* encodes a subunit of ATP-citrate lyase specifically expressed in the tapetum, and a mutation in this gene triggers early tapetal degradation and pollen abortion [21]. Mutants of *dtc1* and *saw1* exhibit enlarged, persistent tapetal and middle-layer cells with delayed degradation [22,23]. These genes function within an interconnected regulatory hierarchy: TDR is regulated by UDT1 and interacts with TIP2 to co-regulate *EAT1* expression, thereby fine-tuning PCD. Both OsMS188 and TIP3 interact with TDR, and OsMS188 directly targets downstream genes such as *PTC1* and *EAT1*. OsEDM2L interacts with TIP2 and TDR, influencing methyladenosine (m^6^A) modification of *EAT1* transcripts and thereby affecting the timing of tapetal PCD.

In this study, we identified a novel male-sterile mutant, *nms1* (*non-pollen male sterility 1*), obtained from the ethyl methanesulfonate (EMS)-mutagenized library of the elite indica restorer line Shuhui 498 (R498). We systematically characterized its genetic characteristics, phenotypic traits, cytological features, RNA-Seq and gene expression. Gene mapping, co-segregation analysis, and genetic complementation confirmed that *nms1* is a novel allele of *OsSTRL2*, which encodes a strictosidine synthase-like protein. The non-pollen male sterility phenotype of *nms1* plants is caused by a single amino acid substitution of Val-212 with Glu. These results lay a foundation for the mechanistic understanding of *NMS1*-mediated regulation of tapetal degradation and microspore development and provide valuable genetic resources and theoretical references for the innovation of male-sterile lines and hybrid rice breeding.

## 2. Materials and Methods

### 2.1. Plant Materials

The male-sterile mutant, named *nms1* (*non-pollen male sterility 1*), was isolated from an EMS-mutagenized population of the elite indica restorer line R498. After three generations of backcrossing and self-pollination, the male sterility phenotype exhibited stable inheritance. Rice plants were grown in the paddy fields of the Sichuan Academy of Agricultural Science in Chengdu (Xindu) and Hainan (Yingzhou), respectively. Tobacco (*Nicotiana benthamiana*) plants were cultivated in artificially regulated climate chambers.

### 2.2. Experimental Methods

#### 2.2.1. Phenotypic Characterization

At the heading stage, wild-type (WT) and *nms1* mutant plants with uniform developmental status were randomly uprooted from the field, and their whole-plant growth phenotypes were photographed. Mature spikelets were then randomly selected for detailed morphological imaging, including photographs of intact spikelets, dehulled spikelets (with lemma and palea removed), and anther structures.

For cytological analysis, at the heading stage, anthers from both WT and *nms1* mutant plants were dissected onto glass slides, stained with 1% iodine–potassium iodide (I_2_-KI) solution, and gently pressed with forceps to release pollen grains. Coverslips were applied, and pollen morphology and staining characteristics were observed under a bright-field optical microscope. For each material, 5 spikelets per plant (collected from the upper, middle, and lower portions of the panicle) across 10 plants, with 3 anthers per spikelet (total 150 anthers), were used for pollen fertility examination; the representative microscopic fields were displayed in the results. For the complementation materials, the same method was used for pollen fertility analysis.

#### 2.2.2. Semi-Thin Sectioning of Anthers

Rice panicles of WT and *nms1* mutants, covering anther developmental Stages 7–12, were collected from experimental fields. After removing the leaves and sheaths, the samples were immediately fixed in Carnoy’s solution I (3:1 [*v*/*v*] anhydrous ethanol:glacial acetic acid) and stored at 4 °C. Spikelets at specific developmental stages were subsequently dehydrated, embedded in Technovit 7100 resin, and polymerized at 60 °C for 24 h. Semi-thin sections (10 μm thick) were obtained using a Leica RM2255 rotary microtome (Leica Microsystems, Wetzlar, Germany), stained with 0.1% toluidine blue O (pH 6.8), and observed under a bright-field microscope (Olympus BX53, Olympus, Tokyo, Japan). Representative images of anther cross-sections were captured at 200× magnification.

#### 2.2.3. Construction of the Mapping Population and Genetic Analysis

The *nms1* mutant was used as the female parent and crossed with the wild-type R498 and R3551 to generate F_1_ hybrid seeds. F_1_ plants were subsequently self-pollinated to produce F_2_ seeds, and F_2_ individuals were planted for segregation analysis. Fertility phenotypes of F_1_ plants and statistical analysis of the fertile-to-sterile segregation ratio in the F_2_ population were performed to elucidate the genetic basis underlying male sterility. The F_2_ population generated from *nms1*/R3551 was used for map-based cloning, and that from *nms1*/R498 was employed for resequencing-based gene mapping.

#### 2.2.4. DNA Extraction and Gene Mapping

In the *nms1*/R3551 F_2_ population, 328 male-sterile individuals were randomly selected as the mapping population. Genomic DNA was extracted from leaf tissues using the cetyltrimethylammonium bromide (CTAB) method. To initially delineate the genomic region harboring the male sterility gene, preliminary mapping was performed using simple sequence repeat (SSR) and insertion–deletion (InDel) markers distributed across all 12 rice chromosomes, which were identified between the *nms1* mutant and R3551. Genomic DNA was extracted from an equal-amount leaf pool consisting of 30 male-sterile individuals from the *nms1*/R498 F_2_ population, as well as from wild-type individuals with normal fertility. All DNA samples underwent quality control and were then sent to Novogene Co., Ltd. (Beijing, China) for whole-genome resequencing using an Illumina HiSeq platform. Gene mapping was performed using a bulked segregant analysis (BSA)-based whole-genome resequencing approach. The primers used for gene mapping are listed in Table S1.

#### 2.2.5. Analysis of Candidate Genes

Candidate gene analysis was performed using the integrated results of preliminary molecular marker mapping and BSA-based gene mapping. The R498 genome, obtained from the Molecular Breeding Knowledgebase (MBKbase, https://www.mbkbase.org/rice, accessed on 15 August 2025), was used as the reference genome for sequence alignment analyses. Based on preliminary gene mapping using polymorphic molecular markers, candidate genes completely linked to the male-sterile trait were screened. Specific sequencing primers were designed to target the predicted mutation sites in the candidate genes. This was followed by polymerase chain reaction (PCR) amplification and sequencing to compare the genotypic differences between wild-type plants and *nms1* mutants. All primer synthesis and sequencing services were provided by Beijing TsingKe Biotech Co., Ltd., Chengdu Branch (Chengdu, China).

#### 2.2.6. Phenotype–Genotype Co-Segregation Analysis

A total of 120 fertile and 120 sterile individuals were randomly sampled from the *nms1*/R498 F_2_ segregating population, and genomic DNA was isolated from the fresh leaves of each selected plant using the CTAB method. Based on the mutation site in the candidate gene, a derived cleaved amplified polymorphic sequence (dCAPS) marker was designed and utilized for PCR amplification. The amplified products were subsequently digested with the restriction enzyme *BglIII* (Figure S1) to analyze the co-segregation between the male sterility phenotype and the candidate gene locus. Subsequently, specific sequencing primers (Table S1) were used for PCR amplification.

#### 2.2.7. RNA Extraction and qRT-PCR Analysis

Tissues (roots, stems, leaves, and anthers at various developmental stages) from WT and *nms1* mutant plants were collected, flash-frozen in liquid nitrogen, and ground to a fine powder. Total RNA was extracted using the RNAprep Pure Plant RNA Extraction Kit (TIANGEN Biotech, Beijing, China). RNA concentration and purity were assessed using a NanoDrop 2000 spectrophotometer (Thermo Fisher Scientific, Waltham, MA, USA). For each sample, 500 ng of total RNA was reverse transcribed into cDNA using the PrimeScript™ RT Reagent Kit with gDNA Eraser (Takara Bio, Dalian, China). Quantitative real-time PCR (qRT-PCR) was performed on a Roche LightCycler^®^ 96 system (Roche Diagnostics, Basel, Switzerland) with One Step PrimeScript™ III RT-qPCR Mix (Takara Bio, Kusatsu, Japan). *Actin1* gene (*LOC_Os03g50885*) was used as the internal reference. Each sample was analyzed with three biological replicates and four technical replicates. Data are presented as mean ± SD of biological replicates. Differences in expression between wild-type and *nms1* plants were considered significant when *p* < 0.01 (probability level). Relative gene expression levels were calculated using the 2^−ΔΔCt^ method. Primer sequences for qRT-PCR are listed in Table S1.

#### 2.2.8. Functional Complementation and Subcellular Localization Vector Construction

For the functional complementation test, the full-length genomic fragment of *NMS1*, including the promoter region, coding sequence (CDS), and 3′-untranslated region (3′-UTR), was amplified from R498 genomic DNA by PCR. The amplified product was purified and subsequently inserted into the restriction enzyme-linearized vector *pC1300* through single-fragment homologous recombination to construct the *NMS1-pC1300* complementation vector. The resulting construct was introduced into calli derived from homozygous *nms1* mutant seeds, after genotyping by extracting DNA from the endosperm of F_2_ hybrid seeds. The pollen fertility of each line at the T_0_ generation was examined according to the method of Section 2.2.1.

To investigate the subcellular localization of *NMS1*, the CDS of *NMS1* (with the stop codon removed) was amplified from anther cDNA of R498 by PCR. Following purification, the amplicon was cloned into the linearized *pC2300-eGFP* vector by homologous recombination, generating the fusion expression construct 2×*35S::NMS1-eGFP*. Then, the *Agrobacterium tumefaciens* strain *GV2260* carrying this construct was infiltrated into tobacco leaves. The fluorescence was observed using a confocal laser scanning microscope (Zeiss LSM780, Carl Zeiss Microscopy GmbH, Jena, Germany).

#### 2.2.9. Transcriptome Analysis

Fresh panicles of wild-type plants and *nms1* mutants at the heading stage were collected from field-grown plants, then immediately flash-frozen in liquid nitrogen and stored at −80 °C until processing. Florets at developmental Stages 7–8 and 11–12 were dissected using fine-tipped forceps on dry ice. Three biological replicates were analyzed per genotype, each comprising a pool of anther developmental Stages 7–8 and 11–12. Wild-type and mutant florets were separately aliquoted into 50 mL RNase-free centrifuge tubes and shipped on dry ice to Novogene Co., Ltd. (Beijing, China) for RNA sequencing. Strand-specific mRNA libraries were constructed using the Illumina TruSeq Stranded mRNA Library Prep Kit and sequenced on the Illumina NovaSeq 6000 platform (Illumina, Inc., San Diego, CA, USA) with 150 bp paired-end reads. An average of approximately 50 million clean reads were generated per sample, with over 90% of bases reaching a Q30 quality score after quality filtering; approximately 92% of the clean reads were successfully mapped to the R498 reference genome, and gene expression levels were quantified as fragments per kilobase of transcript per million mapped reads (FPKM). Differentially expressed genes (DEGs) were identified using DESeq2 with the criteria |log_2_^FoldChange^| ≥ 1 and adjusted *p*-value (Benjamini–Hochberg’s false discovery rate [FDR]) < 0.05. The raw RNA-seq reads have been deposited in the NCBI Sequence Read Archive (SRA) database under accession number PRJNA1512152, and this information has been incorporated into the Data Availability Statement. Transcriptome analysis focused on Gene Ontology (GO) and Kyoto Encyclopedia of Genes and Genomes (KEGG) enrichment analyses, following the bioinformatics pipeline provided by Novogene Co., Ltd. (Beijing, China).

## 3. Results and Analysis

### 3.1. Phenotypic Analysis of the Nms1 Mutant

**Figure 1:**
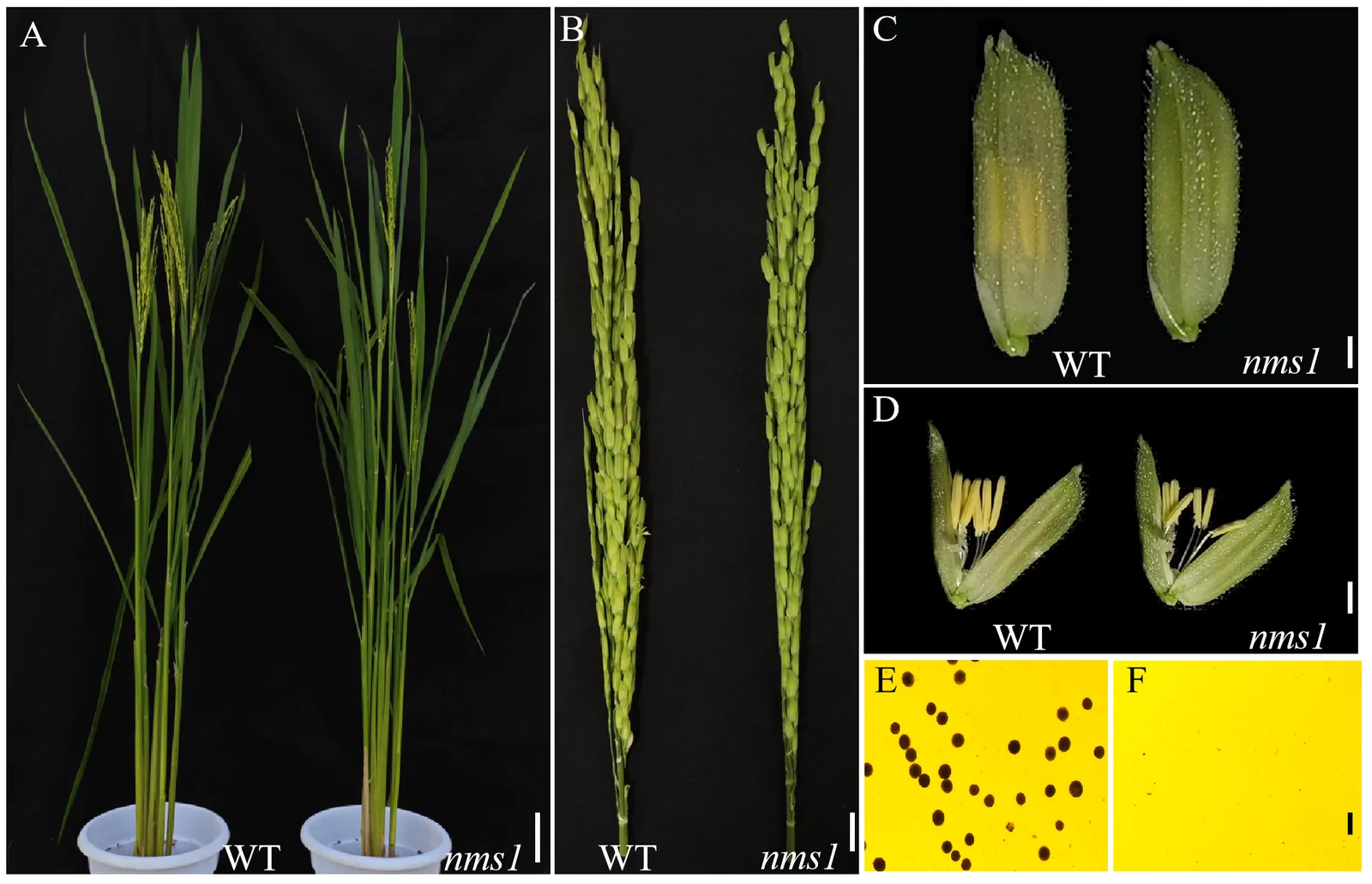
Phenotypic comparison between the *nms1* mutant and wild-type (WT) plants. (**A**) Whole-plant morphology of WT and *nms1* mutant plants (bar = 10 cm). (**B**) Panicle architecture comparison between WT and *nms1* mutant (bar = 3 cm). (**C**,**D**) Mature florets with glumes removed from WT (**C**) and *nms1* mutant (**D**) plants (bar = 2 mm). (**E**,**F**) I_2_-KI-stained mature pollen grains of WT (**E**) and *nms1* mutant (**F**) plants (bar = 50 μm).

A stably inherited male-sterile mutant, *nms1*, was isolated from an EMS-mutagenized population of the *indica* elite restorer line R498. Field observations of *nms1* and WT plants across developmental stages revealed no significant differences in whole-plant morphology or heading timing during the vegetative growth stage (Figure 1A,B). Anatomical examination of spikelets showed that the hull size was slightly smaller in the *nms1* plants (Figure 1C). Upon removal of the lemma and palea, *nms1* plants exhibited fully developed pistils and six stamens per floret. However, compared to the plump, bright-yellow anthers of WT, *nms1* anthers displayed a pale-yellow coloration, reduced size, and elongated filaments (Figure 1D). To assess pollen viability, mature anthers from *nms1* and WT were stained with I_2_-KI solution. Microscopic analysis revealed abundant spherical, densely stained (black) pollen grains in WT anthers (Figure 1E), whereas *nms1* anthers lacked stained pollen grains (Figure 1F). These results demonstrate that *nms1* is a pollenless male-sterile mutant.

### 3.2. Semi-Thin Section Analysis of the Nms1 Mutant Anthers

**Figure 2:**
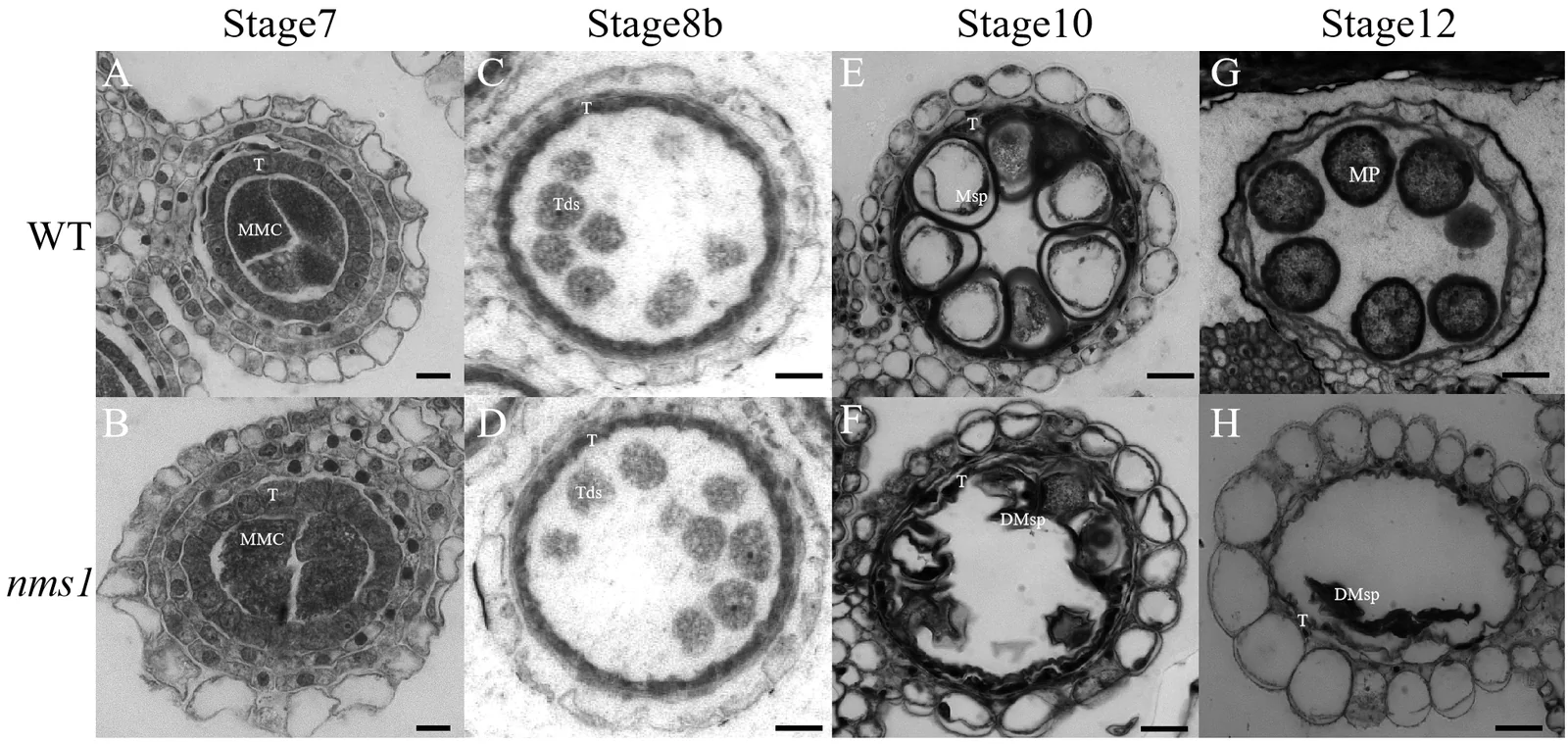
Semi-thin section observations of WT and *nms1* plants. (**A**,**C**,**E**,**G**) WT anther semi-thin sections. (**B**,**D**,**F**,**H**) *nms1* mutant anther semi-thin sections. (**A**,**B**) Anther developmental Stage 7. (**C**,**D**) Anther developmental Stage 8b. (**E**,**F**) Anther developmental Stage 10. (**G**,**H**) Anther developmental Stage 12. T: tapetum; MMC: microspore mother cell; Tds: tetrads; Msp: microspore; DMsp: degenerated microspore; MP: mature pollen. Bar = 20 μm.

To investigate the cause of anther abortion in *nms1*, semi-thin cross-sections of WT and *nms1* anthers at distinct developmental stages were analyzed. At Stage 7, no morphological differences were observed between *nms1* and WT anthers (Figure 2A,B). Both exhibited four well-defined anther wall layers—epidermis, endothecium, middle layer, and tapetum—enclosing the microspore mother cells (MMCs) within the locule. By Stage 8b, MMCs in WT completed meiosis, forming tetrads, while the tapetum displayed condensed and tightly arranged cells (Figure 2C,D). Before Stage 10, *nms1* anther development appeared normal. However, at Stage 10, significant divergence emerged (Figure 2E,F): WT microspores became vacuolated and spherical, accompanied by tapetal degradation associated with altered PCD timing, whereas *nms1* microspores exhibited shrinkage and malformation, with persistent undegraded tapetal residues. By Stage 12, WT microspores matured into pollen grains, filling the locular cavity, while *nms1* microspores degenerated into collapsed structures, and remnants of unprocessed tapetal cells adhered to the inner anther wall (Figure 2G,H). These findings suggest that delayed tapetal degradation associated with altered PCD timing in *nms1* disrupts microspore development, ultimately leading to pollen abortion.

### 3.3. Genetic Analysis of Nms1

We crossed *nms1* plants with R498 and R3551, respectively, to generate F_1_ progeny. All F_1_ plants displayed normal fertility, suggesting that the sterility phenotype in *nms1* is recessive. Subsequent self-pollination of these F_1_ plants yielded F_2_ populations that were segregated for fertility. Statistical analysis using the chi-square (χ^2^) test confirmed a 3:1 fertile-to-sterile ratio in both F_2_ populations (Table 1), consistent with the Mendelian segregation ratio for a single-gene trait. Genetic analysis confirmed that the male sterility observed in *nms1* is conferred by a recessive allele at a single nuclear locus.

### 3.4. Genetic Mapping of Nms1

**Figure 3:**
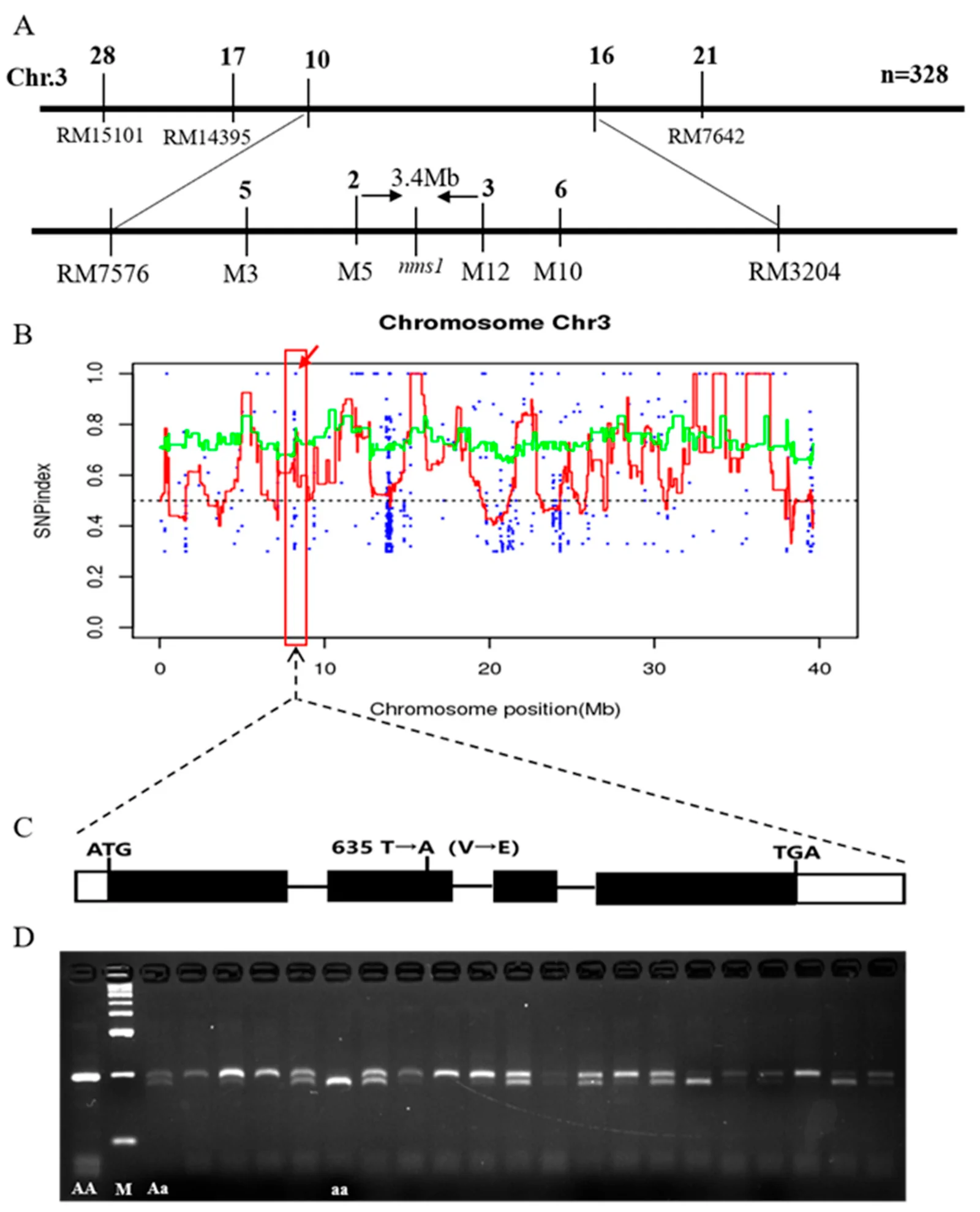
Molecular mapping and candidate gene analysis of the *NMS1* gene. (**A**) Primary mapping of the *NMS1* gene between the InDel markers M5 and M12 on the long arm of chromosome 3. (**B**) Mapping of *NMS1* by BSA-based whole-genome resequencing analysis. The red lines indicate SNP index values for the high trait-value bulk, green lines indicate SNP index values for the low trait-value bulk, and blue dots indicate raw SNP index values for individual SNP loci. (**C**) Structure of *OsR498G0305626400.01*. Black boxes indicate exons, white boxes indicate UTRs, and black lines indicate introns. (**D**) Results of dCAPS marker analysis. M indicates the DNA ladder for agarose gel electrophoresis. AA, Aa, and aa indicate the homozygous WT, heterozygous, and homozygous mutant genotypes, respectively.

To map the *NMS1* gene, 120 polymorphic SSR markers and 16 InDel molecular markers were used for genetic mapping. Linkage analysis using these markers narrowed a 3.4-Mb physical interval between the InDel marker M5 (at 7.86 Mb) and the InDel marker M12 (at 11.29 Mb) on the long arm of chromosome 3 (Figure 3A).

### 3.5. Candidate Gene Sequence Analysis

To clone the *NMS1* gene, we performed an integrated analysis based on preliminary mapping data using a BSA-based whole-genome resequencing approach (Figure 3B). Alignment to the R498 reference genome revealed a single SNP that perfectly co-segregated with the mutant phenotype (SNP index = 1) within the initially mapped 3.4-Mb genomic region. This SNP was located in the *OsR498G0305626400.01* gene, which encodes a strictosidine synthase (Table S2). PCR amplification and sequencing analysis of the genomic sequence of the *OsR498G0305626400.01* gene from WT and *nms1* mutants revealed a T-to-A substitution at position 635 in the coding region of the second exon, resulting in a missense mutation (Val212Glu), where valine (Val) at residue 212 was replaced by glutamic acid (Glu) (Figure 3C and Figure S2). Based on dCAPS marker genotyping, DNA sequencing, and co-segregation analysis of fertility traits in the F_2_ population, we confirmed that all male-sterile plants were homozygous for the mutation. Conversely, all normal fertile plants were either homozygous or heterozygous for the wild-type allele (Figure 3D and Figure S3). Additionally, the mutation segregated in a 1:2:1 ratio in the F_2_ population (Table 1). Based on these results, *OsR498G0305626400.01* was selected as the candidate gene for *NMS1*.

### 3.6. Functional Complementation of Nms1

**Figure 4:**
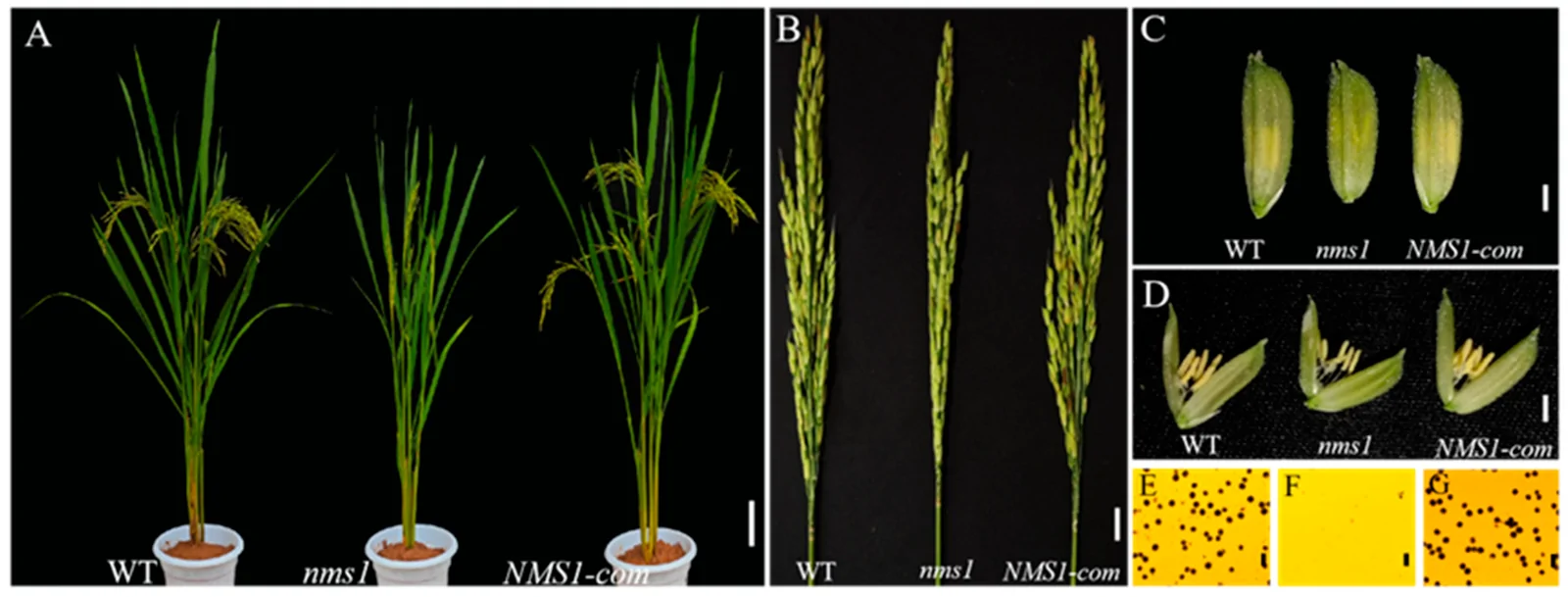
Functional complementation verification of *NMS1*. (**A**) Whole plants of WT, *nms1* mutant, and the complemented line (*NMS-com*). (**B**) Panicles of WT, *nms1*, and the complemented line (*NMS-com*) at the heading stage. (**C**) Spikelets of WT, *nms1,* and the *NMS-com* line. (**D**) Anthers of WT, *nms1*, and the complemented line (*NMS-com*). (**E**–**G**) I_2_-KI-stained mature pollen grains of the wild-type plant, *nms1* mutant, and the complemented line (*NMS1-com*). Bar = 10 μm (**A**); 3 cm (**B**); 2 mm (**C**,**D**); 50 μm (**E**–**G**).

To further verify that *OsR498G0305626400.01* is the causal gene for *NMS1*, which controls male fertility, a functional complementation assay was performed. Phenotypic and molecular characterization of positive transgenic plants (*NMS1-com*) demonstrated that their overall architecture during the vegetative growth stage was comparable to that of WT plants (Figure 4A). In addition, no obvious differences were observed in panicle morphology or spikelet structure compared with the WT (Figure 4B,C). Anthers exhibited normal morphology and coloration (Figure 4D), and pollen fertility was almost fully recovered, as confirmed by I_2_-KI staining (16 out of 20 plants were fertile) (Figure 4E–G). These results demonstrate that the male sterility phenotype in the *nms1* mutant is indeed caused by dysfunction of *OsR498G0305626400.01*/*NMS1*, underscoring the essential role of *NMS1* in anther development.

### 3.7. Spatiotemporal Expression Profiling and Subcellular Localization of Nms1

**Figure 5:**
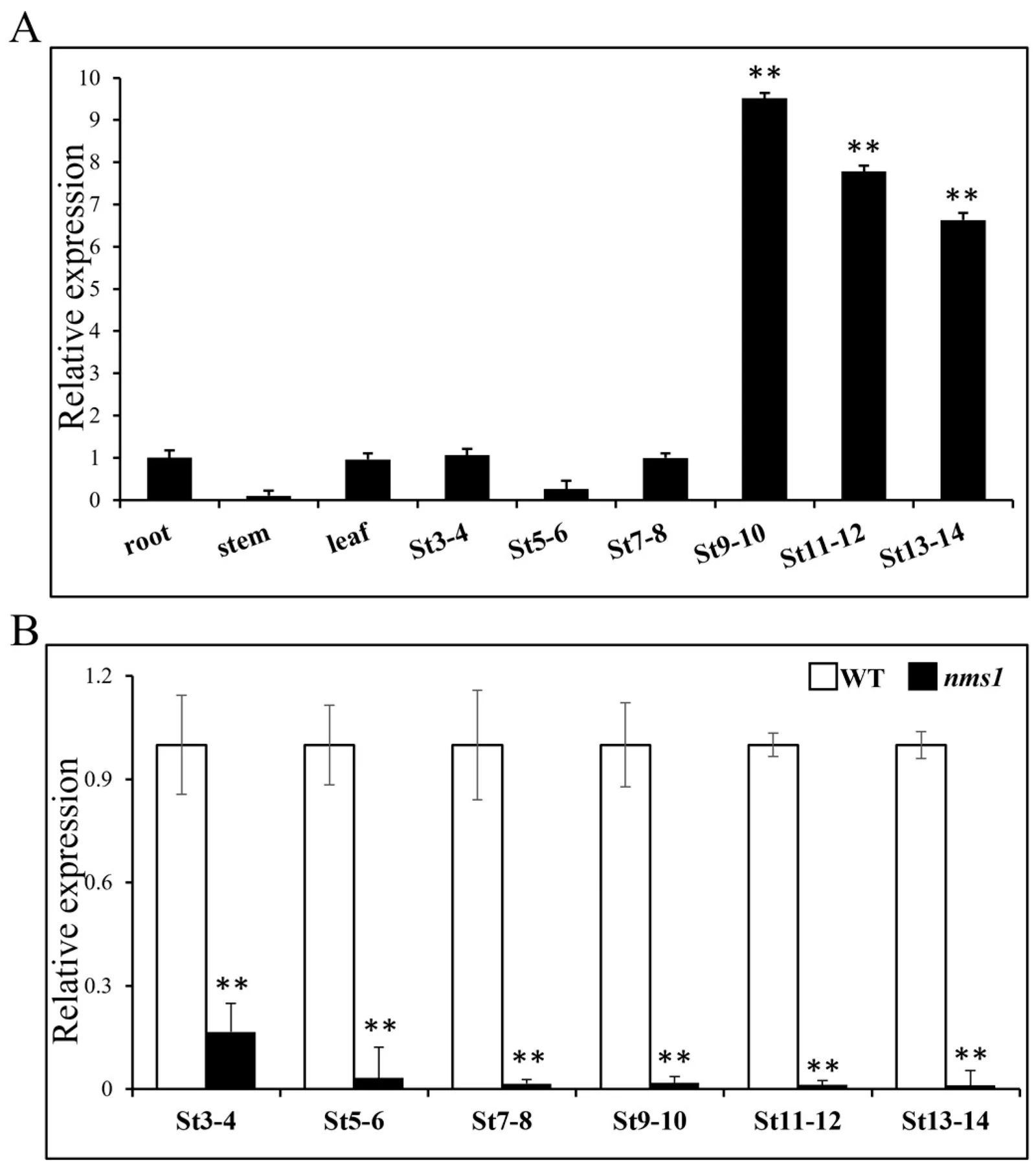
Expression pattern of the *NMS1* gene. (**A**) qRT-PCR analysis of *NMS1* expression in roots, stems, leaves, and spikelets at anther developmental Stages 3–4, 5–6, 7–8, 9–10, 11–12, and 13–14. Expression levels in panel (**A**) are normalized to root tissue (set as 1). (**B**) qRT-PCR analysis of *NMS1* expression in the anthers of WT and *nms1* mutant plants at Stages 3–4 to 13–14. Data are presented as mean ± SD of three biological replicates. In (**A**), double asterisks ** indicate statistically significant differences compared with root tissue at *p* < 0.01; in (**B**), double asterisks ** indicate statistically significant differences between WT and nms1 at the same developmental stage at *p* < 0.01. Statistical significance was determined by two-tailed Student’s *t*-test.

To investigate the transcriptional expression dynamics of the *NMS1* gene, qRT-PCR was performed using tissues from heading-stage rice plants, including roots, stems, leaves, and spikelets at anther developmental Stages 3–4, 5–6, 7–8, 9–10, 11–12, and 13–14. *NMS1* exhibited high transcript abundance in spikelets at Stages 9–10, 11–12, and 13–14, with the highest expression at Stages 9–10 (Figure 5A). In contrast, its expression was negligible in vegetative tissues (roots, stems, leaves) and minimal in spikelets at Stages 5–6. Comparative analysis of *NMS1* expression in WT and *nms1* anthers revealed a significant reduction in transcript levels across all developmental stages in the mutant (Figure 5B). These results indicate that *NMS1* is specifically upregulated during late anther development and is functionally critical for this process. To determine the subcellular localization of NMS1 protein, a C-terminally fused GFP construct under the cauliflower mosaic virus 2×*35S* promoter (*2*×*35S::NMS1-GFP*) was transiently expressed in tobacco epidermal cells. Confocal microscopy detected GFP fluorescence predominantly in endoplasmic reticulum (ER)-enriched ring structures (Figure S4). These findings demonstrate that NMS1 localizes to the ER, suggesting its functional role in ER-mediated regulatory pathways during rice anther development.

### 3.8. Transcriptomic Profiling of NMS1

**Figure 6:**
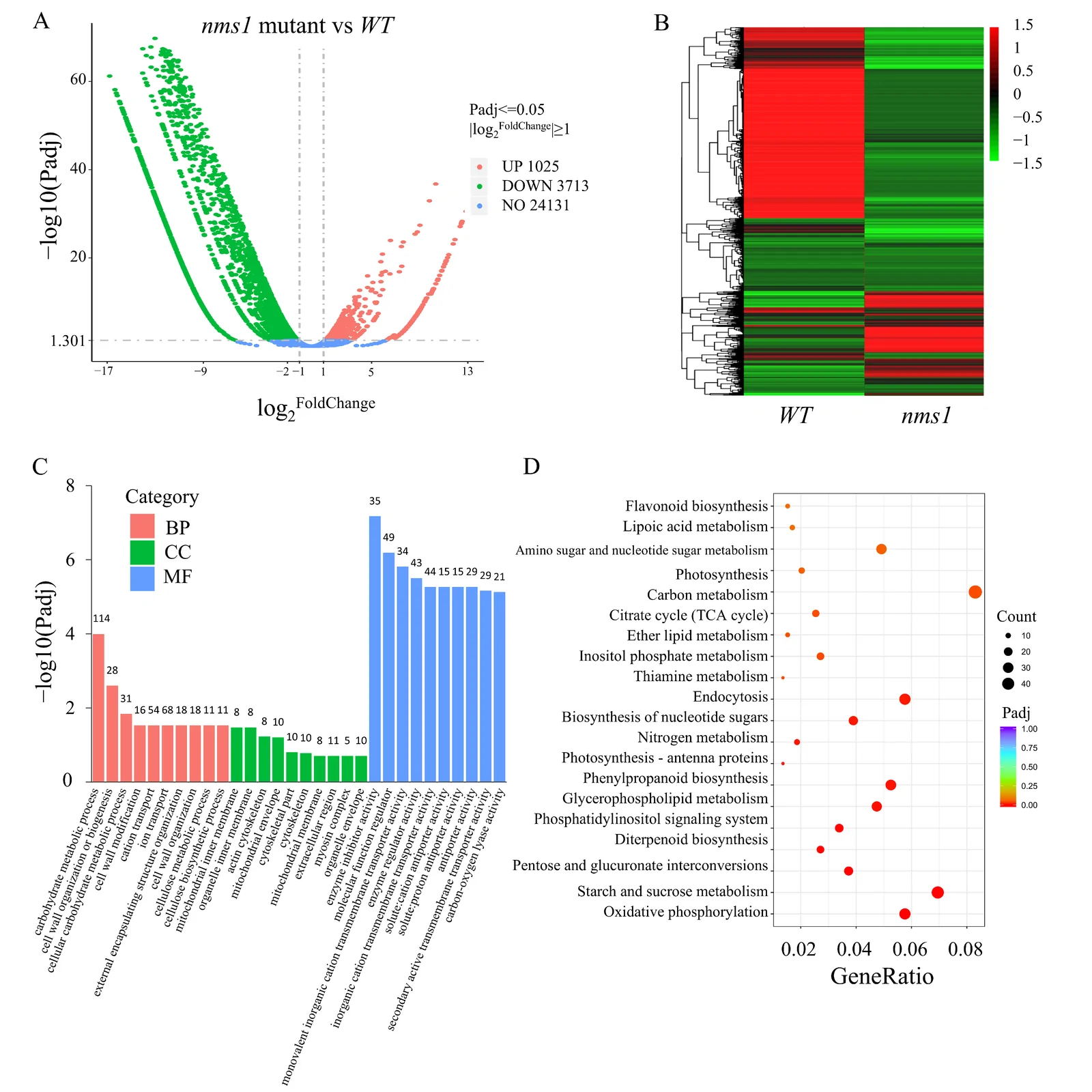
Transcriptomic data analysis of the *nms1* mutant. (**A**) Volcano plot of differentially expressed transcripts (|log_2_^FoldChange^| ≥ 1; Benjamini–Hochberg’s adjusted *p*-value < 0.05) between the WT and *nms1* plants. (**B**) Cluster diagram of differentially expressed genes between the WT and *nms1* plants. (**C**) Gene Ontology (GO) enrichment analysis of the differentially expressed genes. (**D**) Kyoto Encyclopedia of Genes and Genomes (KEGG) pathway enrichment analysis of differentially expressed genes. RNA-seq was performed using three biological replicates per genotype. DEG identification criteria: genes with |log_2_^FoldChange^| ≥ 1 and Benjamini–Hochberg’s adjusted *p* < 0.05 were considered differentially expressed.

To elucidate the role of *NMS1* during late anther development in rice, RNA sequencing (RNA-seq) was performed on spikelets from WT and *nms1* mutants at anther developmental Stages 11–12. Comparative transcriptome analysis revealed that *NMS1* dysfunction was associated with altered expression of 4738 genes (DEGs, Differentially Expressed Genes, |log_2_^FoldChange^| ≥ 1, Benjamini–Hochberg’s FDR < 0.05), including 3713 downregulated and 1025 upregulated transcripts (Figure 6A,B). The GO functional enrichment analysis showed that the DEGs were mainly enriched in biological processes (BP, Biological Process, including carbohydrate metabolism, cell wall organization, ion transport, etc.), cellular components (CC, Cellular Component, including mitochondrial inner membrane, cytoskeleton, myosin complex, etc.), and molecular functions (MF, Molecular Function, including enzyme regulatory activity, molecular function regulator, transmembrane transporter activity, etc.) (Figure 6C, Table S3). KEGG enrichment analyses revealed that several import pathways were significantly perturbed in the *nms1* mutant, including oxidative phosphorylation, starch and sucrose metabolism, pentose and glucuronate interconversions, glycerophospholipid metabolism, and phenylpropanoid biosynthesis (Figure 6D, Table S4). Further interrogation of DEGs mapped to the aforementioned enriched terms revealed that 26 genes associated with rice anther and pollen growth and development were significantly dysregulated in the *nms1* mutant relative to the WT (Table S5, Figure S5). These genes primarily contribute to regulating tapetal degradation associated with PCD timing, mediating energy and substance supply, facilitating sporopollenin deposition, and governing pollen wall development. Their normal expression is closely linked to proper anther development in rice. The significant alterations in their expression levels in the *nms1* mutant suggest that *NMS1* may play a critical role in tapetal degradation and pollen development in rice.

### 3.9. Expression Analysis of Tapetum and Pollen Development-Related Genes

**Figure 7:**
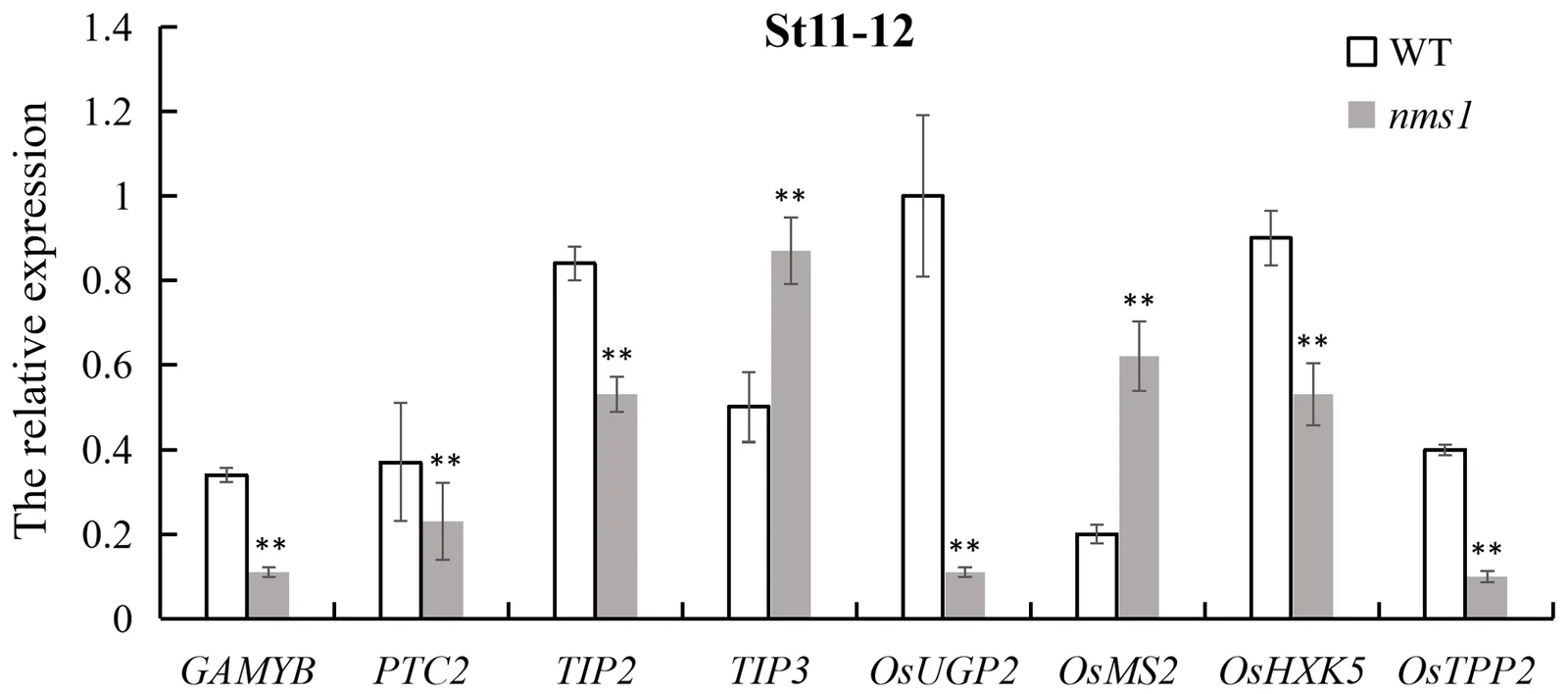
Expression analysis of tapetum and pollen development-related genes in the WT and *nms1* plants. Anthers at developmental Stages 11–12 of WT and *nms1* mutant were used for analysis. Each sample comprised three independent biological replicates. Data are presented as mean ± SD. ** indicates a significant difference at *p* < 0.01, determined by a two-tailed Student’s *t*-test.

The development of anthers and pollen is regulated by multiple genes, such as *GAMYB*, *PTC2*, *TIP2*, *TIP3*, *OsUGP2*, *OsMS2*, *OsHXK5* and *OsTPP2*, several of which were identified by RNA-seq. To validate the transcriptome sequencing results and further investigate genes that potentially function downstream of *NMS1*, we performed qRT-PCR analysis on these genes (Figure 7). The results demonstrated that six genes, *GAMYB*, *PTC2*, *TIP2*, *OsUGP2*, *OsHXK5*, and *OsTPP2* exhibited significantly downregulated expression in the *nms1* mutant compared to WT during Stages 11–12 of anther development. In contrast, *TIP3* and *OsMS2* showed upregulation in the *nms1* mutant compared to WT. These expression patterns were consistent with the transcriptomic data and supported that the aforementioned genes may be downstream targets of *NMS1* and, under its direct or indirect regulation, cooperatively regulate anther and pollen development in rice.

## 4. Discussion

### 4.1. NMS1 Is a Key Regulator of Rice Male Fertility

Phenotypic analysis of the *nms1* mutant showed no observable defects compared to the WT during the vegetative growth stage. However, at the reproductive stage, the mutant exhibited complete male sterility. Cytological observation of anther semi-thin sections indicated severe developmental defects in *nms1* anthers, primarily manifested as substantial malformation and developmental arrest of microspores, leading to complete pollen abortion. The tapetum, as the innermost somatic layer of the anther wall, plays an essential role in pollen development by providing both regulatory and supportive functions. It precisely secretes key nutrients (e.g., sugars, amino acids, Mg^2+^) and sporopollenin precursors into the anther locule, providing essential microenvironments for microspore morphogenesis and maturation [25]. During early development, the tapetum synthesizes callose to enclose microspore mother cells and later secretes callase to ensure the timely degradation of callose, facilitating the release of tetrad microspores [26]. Subsequently, the tapetum undergoes a tightly regulated PCD process, and its degradation products (e.g., nucleic acids, proteins) are recycled as nutrients for microspore maturation. At the uninucleate microspore stage (Stage 10), the *nms1* mutant exhibited aberrant microspore morphology, a phenotype consistent with delayed tapetal degradation associated with altered PCD timing. By the pollen maturation stage (Stage 12), undegraded tapetal debris accumulated prominently in the anther locules. These findings suggest that the fundamental defect in the *nms1* mutant may be the dysregulated tapetal degradation associated with altered PCD timing. This dysregulation led to multiple developmental defects: compromised tapetal integrity disrupted the synthesis and targeted transport of nutrients and sporopollenin precursors; persistent tapetal residues physically impeded microspore development; and delayed catabolic processes interrupted nutrient supply. Together, these disruptions impaired microspore morphogenesis and maturation, ultimately resulting in pollen abortion and complete male sterility in the *nms1* mutant.

### 4.2. NMS1 Encodes the Strictosidine Synthase Protein

By genetic mapping, we identified a critical point mutation in the second exon of the *OsR498G0305626400.01* gene on chromosome 3: a T-to-A substitution at position 635, resulting in a missense mutation where valine at residue 212 was replaced by glutamic acid in the encoded protein (Val212Glu). Sequence alignment of the NMS1 protein between the WT and *nms1* plants revealed that the mutation site is located within the strictosidine synthase domain (Figure S6). Genomic sequencing of multiple *nms1* mutant individuals confirmed the uniformity of this mutation. Genetic linkage analysis demonstrated that this mutation co-segregated with the male sterility phenotype. Moreover, functional complementation assays with the full-length *OsR498G0305626400.01* genomic fragment fully restored male fertility in *nms1* mutants. Taken together, the precise genetic mapping, strict genotype–phenotype co-segregation, and successful functional complementation assays indicated that we identified *OsR498G0305626400.01* as *NMS1,* the gene responsible for male sterility in the *nms1* mutant, and that the T-to-A substitution at position 635 in the *NMS1* gene plays a key role in regulating pollen sterility. *NMS1* is also designated as *OsSTRL2* and encodes strictosidine synthase [26], whereas *OsSTRL2* has been linked to tapetal programmed cell death and pollen wall formation in previous work. The present study identifies a new *nms1* allele in the R498 indica background and demonstrates that a Val212Glu substitution within the strictosidine synthase domain is sufficient to cause male sterility. A multiple-sequence alignment of *NMS1* orthologs from representative plant species—*O. sativa* (*japonica* and *indica*), *Zea mays*, *Sorghum bicolor*, *Setaria viridis*, *Brachypodium distachyon*, *Triticum aestivum*, and *Arabidopsis thaliana*—shows that Val212 is highly conserved (present in 6 of 7 species; the only substitution, Ile in *Arabidopsis*, is a conservative hydrophobic replacement) and located within the invariant RNGS[V/I]FFTD motif of the strictosidine synthase-like domain, supporting its functional importance. In addition, we performed a structural-impact prediction based on the AlphaFold-predicted structure of the *O. sativa indica NMS1* ortholog (UniProt B8BEY4; pLDDT = 98.9 at the Val212-equivalent position), which indicates that Val212 maps to a buried residue in the protein core. The Val-to-Glu substitution introduces a charge reversal (neutral to negative; Grantham’s distance = 121, classified as a radical substitution) at this buried, structurally constrained position, which is predicted to disrupt local hydrophobic packing and destabilize the domain’s fold. Strictosidine synthase is a key rate-limiting enzyme in the terpenoid indole alkaloid (TIA) biosynthetic pathway, catalyzing the condensation of tryptamine and secologanin to form strictosidine. It is primarily expressed in active meristematic tissues and is closely associated with cell division and the development of young plant tissues. Our results demonstrate that the *NMS1*-encoded strictosidine synthase is essential for rice anther and pollen development, and that the valine at position 212 in the protein is key for strictosidine synthase. Thus, this study solidifies the functional significance of strictosidine synthase in rice reproductive development, highlighting its importance as a key regulator of tapetal PCD and pollen formation.

### 4.3. NMS1 Regulates Tapetum and Pollen Development

Phenotypic comparison between the *nms1* mutant and WT plants suggests that pollen abortion in the *nms1* plants results from abnormal tapetum development associated with altered PCD timing and disrupted pollen maturation. The results of transcriptome sequencing and qRT-PCR analysis revealed significant downregulation of tapetum-related genes in the *nms1* mutant relative to the WT plants, including *OsGAMYB*—an R2R3-type MYB transcription factor, which plays an important role in regulating tapetum development [15]—*PTC2*—whose mutation causes non-degraded tapetum and defective pollen wall formation [27]—and *TIP2*—a bHLH transcription factor interacting with and acting upstream of *TDR*, whose disruption leads to complete male sterility [18]. Additionally, pollen development genes—such as *OsHXK5* [28], *OsUGP2* [29], and *OsTPP2* [30] (the latter three genes influence pollen maturation through sugar signaling and metabolism)—were also downregulated in *nms1* mutants. The coordinated downregulation of these genes implies that *NMS1* dysfunction is associated with altered expression of genes within the tapetum and the pollen development network. In contrast, two pollen wall-related genes—*TIP3* [19] and *OsMS2* [31]—were upregulated in *nms1* mutants relative to the WT plants. This upregulation, validated by qRT-PCR, was negatively correlated with *NMS1* loss-of-function, implying the existence of potential antagonistic regulatory or compensatory mechanisms. These results demonstrate that *NMS1* dysfunction disrupts the expression of key genes essential for tapetal and anther development. Further exploration of their functional interactions will be crucial for fully elucidating the precise regulatory network mediated by *NMS1*.

## 5. Conclusions

This study isolated and characterized a rice male-sterile mutant, *nms1*, which exhibits pollenless, complete male sterility. The *nms1* mutant showed pale-yellow, shrunken anthers, elongated filaments, delayed tapetal degradation, and aberrant microspore development at late stages. Genetic analysis revealed that the *nms1* phenotype is controlled by a single recessive nuclear gene. Through an integrated approach combining BSA with whole-genome resequencing, co-segregation analysis, and genetic complementation assays, we identified *OsR498G0305626400.01* as the *NMS1* gene. A T635A mutation within its coding sequence leads to a substitution of valine with glutamic acid at the 212th residue of the encoded strictosidine synthase, which underlies the observed pollen sterility phenotype. *NMS1* exhibits specific and high expression during the late stages of anther development, indicating its pivotal role in anther and pollen development. Gene expression analysis indicated that *NMS1* loss-of-function perturbs the expression of genes involved in tapetal degradation and pollen wall biosynthesis. Thus, the *NMS1* gene plays an indispensable role in microspore development and pollen fertility in rice. This study reports the cloning and functional characterization of *NMS1*, providing novel insights into the molecular mechanisms of male sterility and a valuable genetic resource for *indica* hybrid breeding programs.

## Figures and Tables

**Table 1:** Genetic analysis of the *nms1* plants in the F_2_ populations.

Cross	Number of Total Plants	Normal Plants	Male Sterility Plants	Theoretical Separation Ratio	*χ* ^2^
*nms1*/R498	370	283	87	3:1	0.436
*nms1*/R3551	1346	1018	328	3:1	0.476

## Data Availability

The original contributions presented in this study are included in the article/Supplementary Materials. Further inquiries can be directed to the corresponding author.

## Supplementary Materials

The following supporting information can be downloaded at: https://www.mdpi.com/article/10.3390/genes17080970/s1, **Figure S1:** Schematic diagram of the dCAPS marker analysis method; **Figure S2:** Sanger sequencing analysis of the mutation site of the candidate gene; **Figure S3:** Co-segregation analysis of the mutation site of the candidate gene; **Figure S4:** Subcellular localization of NMS1 protein. Red arrows indicate the ER location of the NMS1 protein. Bar = 20 μm; **Figure S5:** The expression changes of pollen development-related differentially expressed genes between WT and *nms1* plants; **Figure S6:** The sequence alignment of the NMS1 protein between WT and *nms1* mutant Red frames outline the strictosidine synthase domain; **Table S1:** List of primers used in this study; **Table S2:** BSA analysis of the candidate gene prediction in the mapped region; **Table S3:** The numbers of differentially expressed genes of GO significant enriched terms (*p* < 0.05); **Table S4:** The numbers of differentially expressed genes of KEGG significantly enriched terms (*p* < 0.05); **Table S5:** The pollen development-related differentially expressed genes between WT and *nms1* mutant; **Table S6:** Analysis of NMS1 ortholog sequences in 7 species; **Table S7:** Prediction of physicochemical consequences of the Val→Glu substitution in NMS1 using AlphaFold3.

## Abbreviations

The following abbreviations are used in this manuscript:

PCD	Programmed cell death
EMS	Ethyl methanesulfonate
CTAB	Cetyltrimethylammonium bromide
WT	Wild-type
SSR	Simple sequence repeat
InDel	Insertion–deletion
BSA	Bulked segregant analysis
PCR	Polymerase chain reaction
dCAPS	Derived Cleaved Amplified Polymorphic Sequences
CDS	Coding sequence
UTR	Untranslated region
GO	Gene Ontology
KEGG	Kyoto Encyclopedia of Genes and Genomes
MMCs	Microspore mother cells
SNP	Single nucleotide polymorphism
