# Supplementary material for: Strictosidine Synthase-like Gene NMS1 Is Required for Male Fertility in Rice by Regulating Tapetal Degradation
Source: Genes (Basel). 2026 Aug 19;17(8):970. doi: 10.3390/genes17080970 (PMC13512317; doi:10.3390/genes17080970)
Supplement: Supplementary file 1 [file genes-17-00970-s001.zip › Figures S1–S6.pdf]

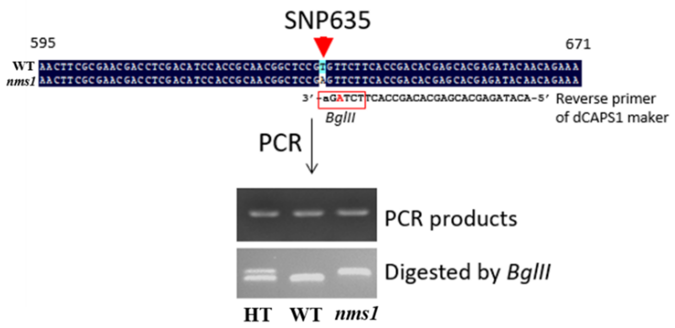

Figure S1 Schematic diagram of the dCAPS marker analysis method

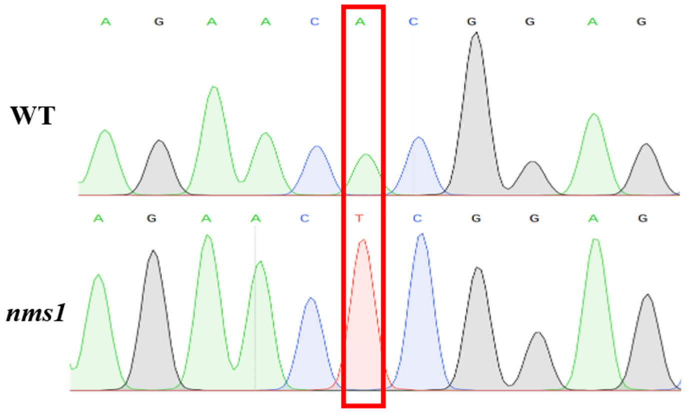

Figure S2 Sanger sequencing analysis of the mutation site of the candidate gene

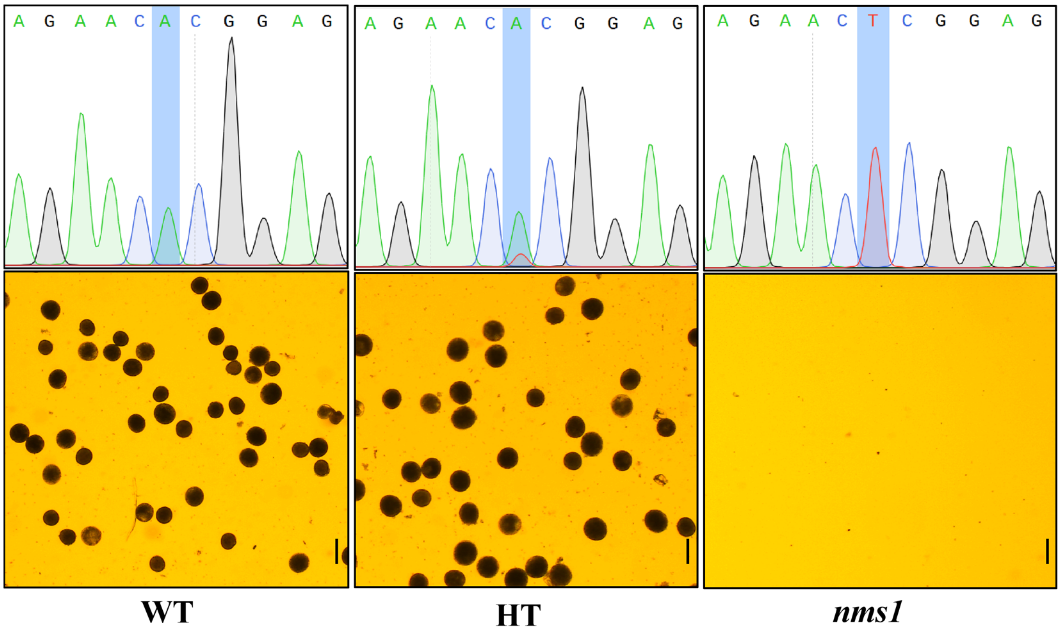

Figure S3 Co-segregation analysis of the mutation site of the candidate gene

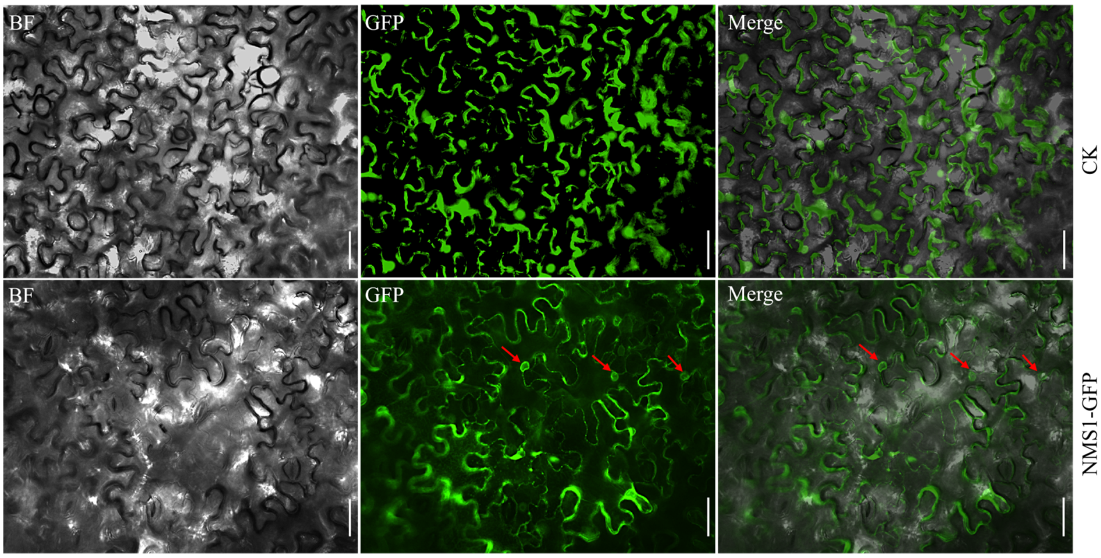

Figure S4 Subcellular localization of NMS1 protein. Red arrows indicate the ER location of the NMS1 protein. Bar = 20 μm.

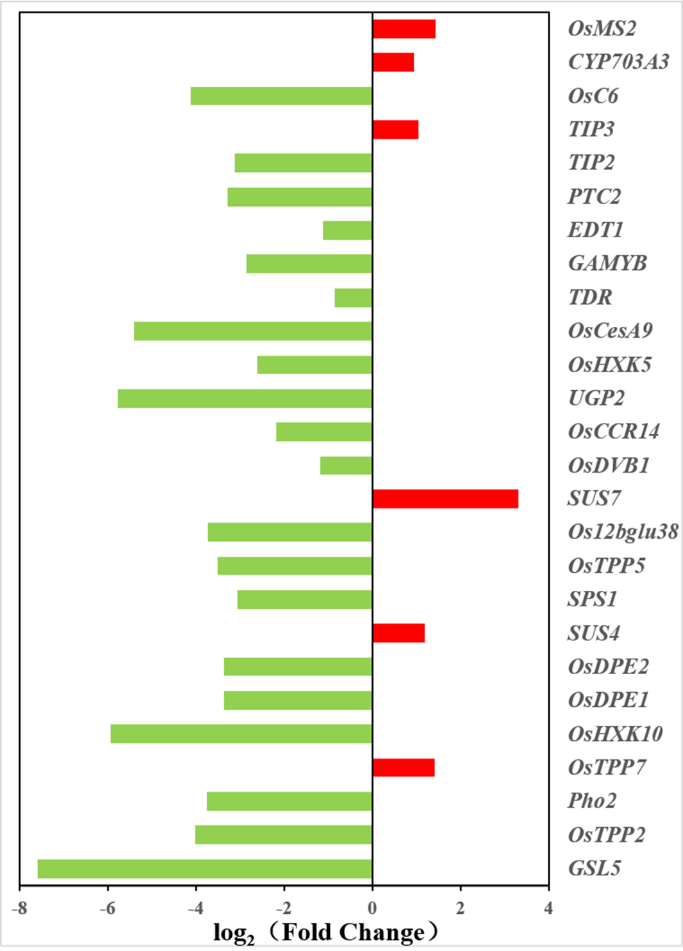

Figure S5 The expression changes of pollen development-related differentially expressed genes between WT and *nms1* plants.

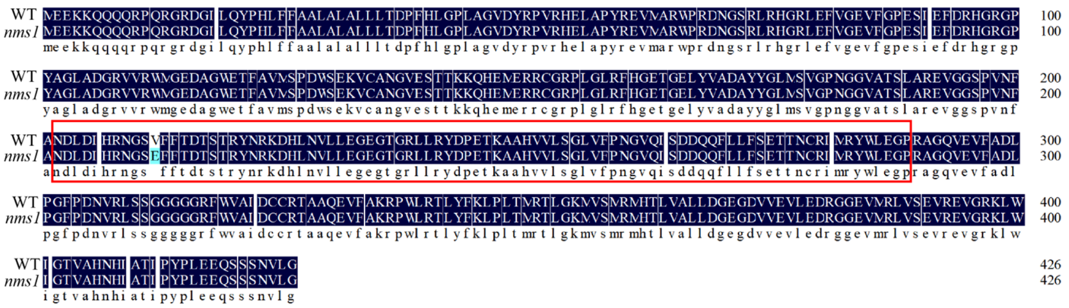

Figure S6 The sequence alignment of the NMS1 protein between WT and *nms1* mutant Red frames outline the strictosidine synthase domain.
