# Supplementary material for: Strictosidine Synthase-like Gene NMS1 Is Required for Male Fertility in Rice by Regulating Tapetal Degradation
Source: Genes (Basel). 2026 Aug 19;17(8):970. doi: 10.3390/genes17080970 (PMC13512317; doi:10.3390/genes17080970)
Supplement: Supplementary file 1 [file genes-17-00970-s001.zip › nms1-Tables S1–S7.pdf]

Table S1 List of primers used in this study

Name	Forward sequence（5'→3'）	Reverse sequence（5'→3'）	Purpose
**RM15101**	TAGTCCTGGTTGGTGATGTCTCTGG	CACAATGTGCACCGTGTAGACAGC	Mapping
**RM14395**	AATATCCGCAGCCGAAACATAGC	ACCGACCAAACCAACACAATCG	Mapping
**RM7576**	GTGGGAAGAAGAACATCAACTGG	GCACACAAGATAAACCCAATCAGC	Mapping
**RM7642**	TTAGATCACGATACTCAGGGATGC	CGAAGAAAGAGAGCACGAAACG	Mapping
**RM3204**	CTTACACACATGGCCCACATGC	TCCTCTCTTCTCACTCTCCCAACC	Mapping
**M3**	TGGAGTAAAGAAAAAGTAT	GTTTGGTAACACCGTATGA	Mapping
**M5**	GATAAAATCAGATAGTGTTGT	TCTGAACAAGGAGAGGATA	Mapping
**M10**	AGGCACGAGGGCTCTACG	GTTCTTCGCCTGCTCCATA	Mapping
**M12**	AAATCAAAATCGTAAAACA	TTCAACTCCACTGCTGCTC	Mapping
nms1-D	ATGGGCAGCTTTGGTTTCTG	GTGAAAAGGGTCCAGGAAAC	Mutation detection
**dCAPS-*nms1***	ACGAAGAAGCAGCACGAGATGGAGCGAC	TGTATCTCGTGCTCGTGTCGGTGAAGATC	Mutation detection
qNMS1-2	CTCCGTGTTCTTCACCGACA	GGAACTGCTGGTCGTCAGAA	qRT-PCR
qGAMYB	GCGACGGTATCATGTTCAAT	GTCGCATAAGAGAACATCTG	qRT-PCR
qPTC2	TTCCATTGGAGGACATGATGAT	AACAAATTAAGTAGCAGCAGCATA	qRT-PCR
qTIP2	AAGGAGGATAGAGCGACGGT	GATGTTCACGTCGTCCTCCA	qRT-PCR
qTIP3	CCGATGACCAGCTGAGGTTT	AGCCCTTCAGCCTGGAATTC	qRT-PCR
qCYP703A3	TGTACTGCTTTCTTTGCCCG	GCAGCAATCATGTCCTGCAT	qRT-PCR
qOsUgp2	CTTTGTTACACGCAACCCGG	GATGGTCACCTTGCCCTTCA	qRT-PCR
qOsMS2	CGGTTCCTCTTCCAGCACTT	GTAGATGGTCTGCTCGACGG	qRT-PCR
qOsHXK5	GGGGTCCCAGATACTTCCCT	ACCCCATATATGCCAGCAGC	qRT-PCR
qOsTPP2	GTGACTGCCGTCCTGAAGAA	GCCAATGCTGTTTGCCTTCA	qRT-PCR
Actin	TGGCATCTCTCAGCACATTCC	TGCACAATGGATGGGTCAGA	qRT-PCR
NMS1-PC1300	tatgaccatgattacgaattcCCGATCACACGAAGACACGA	tctagaggatccccgggtaccGAGTTGTTCTTACTAGTTCC	Vector construction
NMS1-GFP	tggagaggacagggtaccATGGAAGAGAAGAAGCAGCAGC	gtcgactctagaggatccATCACCAAGCACGTTGCTGC	

Table S2 BSA analysis of the candidate gene prediction in the mapped region

Pos	Ref	Alt	Variant	Gene ID	SNP-index	Predicted function
8932320	A	T	nonsynonymous	OsR498G0305626400.01	1.0	Strictosidine synthase

Table S3 The numbers of differentially expressed genes of GO significant enriched terms ($p < 0.05$)

GO_classify1	GO_classify2	DEG number
Biological process	carbohydrate metabolic process	114
	cell wall organization or biogenesis	28
	cellular carbohydrate metabolic process	31
	cell wall modification	16
	cation transport	54
	ion transport	68
	external encapsulating structure organization	18
	cell wall organization	18
	cellulose metabolic process	11
	cellulose biosynthetic process	11
	ion transmembrane transport	26
	polysaccharide metabolic process	21
	carbohydrate biosynthetic process	22
	DNA integration	7
	cation transmembrane transport	23
	inorganic ion transmembrane transport	23
	inorganic cation transmembrane transport	23
	cellular carbohydrate biosynthetic process	19
	response to oxidative stress	26
	cellular glucan metabolic process	18
	glucan metabolic process	18
	cellular polysaccharide metabolic process	18
Cellular component	mitochondrial inner membrane	8
	organelle inner membrane	8
Molecular function	enzyme inhibitor activity	35
	molecular function regulator	49
	monovalent inorganic cation transmembrane transporter activity	34
	enzyme regulator activity	43
	inorganic cation transmembrane transporter activity	44
	solute:cation antiporter activity	15
	solute:proton antiporter activity	15
	antiporter activity	29
	secondary active transmembrane transporter activity	29
	carbon-oxygen lyase activity	21
	proton transmembrane transporter activity	24
	lyase activity	37
	transferase activity, transferring hexosyl groups	79
	cation transmembrane transporter activity	46
	inorganic molecular entity transmembrane transporter activity	53
	ion transmembrane transporter activity	56
	terpene synthase activity	10
	transferase activity, transferring glycosyl groups	90
	carbon-oxygen lyase activity, acting on phosphates	10
	active transmembrane transporter activity	37
	hydrolase activity, hydrolyzing O-glycosyl compounds	68
	hydrolase activity, acting on glycosyl bonds	71
	pectinesterase activity	16
	UDP-glycosyltransferase activity	28
	heme binding	74
	cellulose synthase activity	11
	cellulose synthase (UDP-forming) activity	11
	tetrapyrrole binding	74
	galactoside 2-alpha-L-fucosyltransferase activity	7
	alpha-(1,2)-fucosyltransferase activity	7
	oxidoreductase activity, acting on peroxide as acceptor	29
	antioxidant activity	30
	glucosyltransferase activity	21
	peroxidase activity	28
	fucosyltransferase activity	7

	calcium ion binding	36
	UDP-glucosyltransferase activity	15
	electron transfer activity	26

Table S4 The numbers of differentially expressed genes of KEGG significantly enriched terms ($p < 0.05$)

	Pathway	DEG number
KEGG	Phenylpropanoid biosynthesis	40
	Starch and sucrose metabolism	46
	Pentose and glucuronate interconversions	24
	Oxidative phosphorylation	34
	Diterpenoid biosynthesis	17
	Photosynthesis - antenna proteins	9
	Nitrogen metabolism	12
	Phosphatidylinositol signaling system	20
	Endocytosis	37
	Glycerophospholipid metabolism	28
	Biosynthesis of nucleotide sugars	23

Table S5 The pollen development-related differentially expressed genes between WT and *nms1* mutant

Differentially expressed gene	$\log_2$FoldChange	Putative Function
Os06g0182300 *GSL5*	-7.58	glucan synthase-like 5
Os10g0553300 *OsTPP2*	-4.01	trehalose-6-phosphate phosphatase
Os01g0851700 *Pho2*	-3.75	cytosolic phosphorylase2
Os09g0369400 *OsTPP7*	1.42	trehalose-6-phosphate phosphatase
Os05g0375100 *OsHXK10*	-5.93	hexokinase
Os07g0627000 *DPE1*	-3.37	disproportionating enzyme
Os07g0662900 *OsDPE2*	-3.35	cytosolic disproportionating enzyme 2
Os03g0340500 *SUS4*	1.20	sucrose synthase
Os01g0919400 *SPS1*	-3.05	sucrose phosphate synthase
Os04g0554000 *OsTPP5*	-3.50	trehalose phosphatase
Os12g0420100 *Os12bglu38*	-3.72	β-glucosidase
Os04g0249500 *SUS7*	3.32	sucrose synthase
Os03g0840900 *OsDVB1*	-1.18	decreased vascular bundle 1
Os08g0441500 *OsCCR14*	-2.18	cinnamoyl-CoA reductase
Os02g0117700 *UGP2*	-5.76	UDP-glucose pyrophosphorylase
Os05g0522500 *OsHXK5*	-2.60	hexokinase
Os09g0422500 *OsCesA9*	-5.41	cellulose synthase catalytic subunit
Os02g0120500 *TDR*	-0.84	tapetum degeneration retardation
Os01g0812000 *GAMYB*	-2.86	Gibberellin MYB transcriptional activator
Os11g0696200 *EDT1*	-1.11	ATP-Citrate Lyase
Os02g0820800 *PTC2*	-3.28	AT-hook DNA binding protein
Os01g0293100 *TIP2*	-3.11	bHLH transcription factor
Os03g0716200 *TIP3*	1.05	PHD-finger transcription factor
Os11g0582500 *OsC6*	-4.12	lipid transfer protein
Os08g0131100 *CYP703A3*	0.96	cytochrome P450 hydroxylase
Os03g0167600 *OsMS2*	1.44	fatty acyl carrier protein reductase

Table S6 Analysis of NMS1 ortholog sequences in 7 species

Species	Accession	Length (aa)	Identity to *NMS1* (%)	Residue at Pos. 212
Oryza sativa (japonica) — Reference	XP_015630238.1	427	100	Val
Oryza sativa (indica)	B8BEY4	364	46.2*	Val
Zea mays	AAK52489.1 (ZmMS45)	412	80.6	Val
Sorghum bicolor	KAG0551823.1	412	79.6	Val
Setaria viridis	XP_034574720.1	413	78.5	Val
Brachypodium distachyon	XP_003576476.1	366	40.8	Val
Triticum aestivum	XP_037443443.1	399	42.3	Val
Arabidopsis thaliana	OAP03294.1 (AtLAP3)	414	—	Ile

*Lower identity for indica reflects different isoform/annotation length; pairwise alignment confirmed Val equivalence.

Table S7 Prediction of physicochemical consequences of the Val→Glu substitution in NMS1 using AlphaFold3

Property	Valine (V)	Glutamate (E)	Change
Charge (pH 7)	Neutral	Negative (−1)	Charge reversal
Hydropathy (Kyte–Doolittle)	+4.2 (hydrophobic)	−3.5 (hydrophilic)	Δ = −7.7
Side chain MW	99.13 Da	129.12 Da	+30 Da
Polarity	Non-polar	Polar	Polarity shift
Grantham distance	—	—	121 (radical)

Grantham distance classification: 0–50 conservative, 51–100 moderate, 101–150 radical. Val→Glu at 121 is classified as a radical substitution
